# Synthesis, Characterization and Antitumor Activity of cis-bis(acylthioureato) platinum(II) Complexes, cis-[PtL_2_] [HL^1^=N,N-Diphenyl-N'-Benzoylthiourea or HL^2^=N,N-diphenyl-N'-(p-nitrobenzoyl)thiourea]

**DOI:** 10.1155/S1565363303000219

**Published:** 2003

**Authors:** Wilfredo Hernández, Evgenia Spodine, Juan Carlos Muñoz, Lothar Beyer, Uwe Schröder, Jorge Ferreira, Mario Pavani

**Affiliations:** 1 Universidad de Chile Facultad de Ciencias Químicas y Farmacéuticas Santiago 1 Casilla 233 Chile; 2 Universidad de Chile Facultad de Ciencias Físicas y Matemáticas Santiago 1 Casilla 233 Chile; 3 Universität Leipzig Institut für Anorganische Chemie Johannisallee 29 Leipzig D-04103 Germany; 4 Universidad de Chile Instituto de Ciencias Biomédicas Programa de Farmacología Clínica y Molecular Facultad de Medicina Santiago 1 Casilla 233 Chile

## Abstract

A low-molecular weight chromium-containing fraction of the material resulting from dichromate reduction by bovine liver homogenate was investigated by NMR and ES-MS. The ES-MS spectrum showed a readily detectable peak at m/z 786.1. The same molecular weight reasonably agreed with the relatively low diffusion coefficient measured by NMR-DOSY experiments on the main species observed in the ^1^H NMR spectrum. At least two downfield shifted and broad paramagnetic signals were apparent in the ^1^H NMR spectrum. Temperature dependence of chemical shift was exploited in order to estimate the diamagnetic shift of the signals in the diamagnetic region of the spectrum. 2D TOCSY, NOESY, COSY and ^1^H-^13^C HMQC spectra revealed the presence of aromatic protons (which were assigned as His residues), Gly and some other short chain amino-acids. Combinations of the molecular masses of such components together with acetate (which is present in the solution) and chromium atoms allowed a tentative proposal of a model for the compound.


### Synthesis, Characterization and Antitumor Activity of cis-bis(acylthioureato) platinum(II) Complexes, cis-[$PtL_2$] [$HL^1$ =N,N-Diphenyl-N′-Benzoylthiourea or $HL^2$ = N,N-diphenyl-N′-(p-nitrobenzoyl)thiourea]

Wilfredo Hernández[a], Evgenia Spodine[a,*], Juan Carlos Muñoz[b], Lothar Beyer[c], Uwe Schröder[c], Jorge Ferreira[d] and Mario Pavani[d]

[a]*Universidad de Chile, Facultad de Ciencias Químicas y Farmacéuticas, Casilla 233,Santiago 1, Chile*
[b]*Universidad de Chile, Facultad de Ciencias Físicas y Matemáticas, Casilla 233, Santiago1,Chile.*
[c]*Universität Leipzig, Institut für Anorganische Chemie, Johannisallee 29, D-04103 Leipzig, Germany.*
[d]*Universidad de Chile, Instituto de Ciencias Biomédicas, Programa de Farmacología Clínica y Molecular, Facultad de Medicina, Casilla 233, Santiago 1, Chile.*

### ABSTRACT

Acylthiourea ligands, N,N-diphenyl-N′-benzoylthiourea ($HL^1$ ) and N,N-diphenyl-N′-(p-nitrobenzoyl) thiourea ($HL^2$ ) were prepared in high yields ($HL^1$: 90%, $HL^2$: 82%), by converting benzoyl chloride or p-nitrobenzoyl chloride into the corresponding benzoyl isothiocyanate followed by reacting with diphenylamine. The *cis*-[$PtL_2$] complexes have been synthesized by reaction of the ligands $HL^1$ or $HL^2$ with $K_2PtCl_4$ in a Pt:HL (1:2) molar ratio, in the presence of sodium acetate. The ligands and their *cis*-[$PtL_2$] complexes have been characterized by elemental analysis, IR, FAB(+)-MS,$^1$H-NMR,$^{13}$C-NMR and $^{195}$Pt-NMR. The molecular structure of *cis*-bis(N,N-diphenyl-N′-benzoylthioureato) platinum(II) shows a square-planar geometry with two deprotonated ligands ($L^1$) coordinated to Pt(II) through the oxygen and sulfur atoms in a *cis* arrangement. The antitumor activity of the ligands and their *cis*-[$PtL_2$] complexes was evaluated on mouse mammary adenocarcinoma TA3. The $IC_{50}$.values of culture growth for ligands $HL^1$ and $HL^2$ were 23.1 and 34.9 μM, respectively, whereas for the *cis*-[$PtL_2$] complexes they were in the range of 2.6-2.8 μM, which indicates that the platinum(II) complexes are about 10-fold more cytotoxic than the free ligands and a participation of nitro group in the complex activity is slightly relevant.

**Keywords:** Platinum(II) complexes; Acylthiourea; Antitumor / cytotoxic activity; Cell growth

* Corresponding author. Tel.:+56-2-6782862; fax:+56 -2-678-2868; e-mail: espodine@ciq.uchile.cl

### 1. INTRODUCTION

The transition metal complexes perform an important role in Medicinal Inorganic Chemistry due to their pharmacological properties in survival systems. *Cis*-diaminedichloroplatinum(II) (cisplatin) is an antineoplastic agent of clinical use, highly effective in treating testicular and ovarian cancers /1/. The biological efficiency of cisplatin and its analogues, carboplatin and oxaliplatin /2,3/, as antitumoral drugs is due to the formation of coordination complexes with nuclear DNA, mainly to form Pt-N7 adducts with two adjacent guanine bases (G-G) on the same nucleotide strand /4/. These conformational changes within the DNA double helix originate the inhibition of DNA replication and RNA transcription and produce finally the cell death /5-8/. Despite its efficacy in various neoplastic diseases, cisplatin has several disadvantages due to its propensity to tumor resistance and by causing several types of dose-limiting toxicity, such as, nephrotoxicity, nausea, neurotoxicity and myelosuppression /9,10/. With the possibility of exploring a new class of anticancer agents, Bierbach *et a*l. have synthesized the coordinative saturated platinum(IV) complexes $[PtCl_4(NH_3)L]$ and $[PtCl_3(NH_3)_2L]^+$ (L=1,1,3,3-tetramethylthiourea) by reacting the precursor cisplatin with thiourea derivative ligands. These complexes showed high cytotoxicity *in vitro* at micromolar concentrations against the L1210 leukemia cell of murine /11,12/. Moreover, platinum(II) complexes of type [Pt(en)(L)Cl] (en=ethylenediamine, L= acridinylthiourea) as possible intercalating agents of DNA have been synthesized analogously to cisplatin, by replacing two $NH_3$ groups of cisplatin by one bidentate ligand (en) and a chloride by one monodentate thiourea derivative ligand (L), which coordinates to platinum through a sulfur atom. The cytotoxic activity presented by these complexes was reflected by low values of $IC_{50}$ for HL-60 leukemia cell and human ovarian 2008 and C13 (resistent to cisplatin) cell lines /13/.

In this context, platinum(II) complexes of the type [Pt(L)Cl(DMSO)] (L=acylthiourea ligand, R′-C(O)NHC(S)$NR_2$; R′=aryl, $NR_2$ =amine; DMSO=dimethylsulfoxide) were prepared by Sacht *et al.* for their biological and chemical evaluation /14-16/. The acylthiourea after deprotonation of the amide group (NHCO) can act as a bidentate chelate ligand, coordinating to platinum through the oxygen and sulfur donor atoms. The facility of affording the replacement of the functional groups R and R′ to obtain a wide range of ligands and platinum(II) complexes with different physical and chemical properties, made the assessment of this type of compounds attractive /17,18/. Cytotoxic studies realized on HeLa cancer cell lines have shown that certain platinum(II) complexes with these acylthiourea ligands present cytotoxic behaviour with antiproliferative effects being dependent on the nature or the type of the substituent at the acylthiourea ligand. Moreover, the interaction of [Pt(L)Cl(DMSO)] complexes with nucleotides, calf thymus (ct), poly $[d(A\text{-}T)_2]$ (AT) and poly $[d(G\text{-}C)_2]$ (GC) DNAs has been evaluated indicating conformational changes on DNA /19/.

As a part of our continuing efforts for the synthesis and development of new platinum(II) antineoplastic agents with low toxicity and therapeutic improved action, we have prepared platinum(II) bis-chelate complexes, *cis*-$[PtL_2]$, with acylthiourea ligands, R′-C(O)NHC(S)$NR_2$ , for their cytotoxic evaluation as possible antitumor agents. In this work, we inform of the synthesis and characterization of novel platinum (II) complexes with the ligands N,N-diphenyl-N′-benzoylthiourea ($HL^1$) and N,N-diphenyl-N′-(p-nitrobenzoyl) thiourea ($HL^2$) and their *in vitro* cytotoxic effect on mouse mammary adenocarcinoma TA3.

### 2. EXPERIMENTAL

### 2.1. Materials and measurements

The potassium tetrachloroplatinate ($K_2PtCl_4$) was purchased from Merck, Darmstadt. All other chemicals and solvents (Aldrich) were analytical grade and used as supplied, except the acetone which were distilled before use. Elemental analysis were determined on a Fisons-Carlo Erba 1108 elemental microanalyser. Melting points were determined on a Boetius melting-point apparatus. The infrared (IR) spectra were recorded in solid state (KBr pellets) on a Bruker FT-IR IFS 55 Equinox spectrophotometer in the range 4000 - 400 $cm^{-1}$ . The $^{1}H$ (300 MHz) and $^{13}C$ (75.5 MHz) NMR spectra were recorded on a Bruker advance DRX 300 spectrometer. $^{195}Pt$ -NMR spectra were recorded at 86 MHz on a Bruker DRX 400 spectrometer at 300 K using $CDCl_3$ as solvent. The chemical shifts (δ) were measured relative to TMS in the case $^{1}H$ and $^{13}C$, and to $Na_2PtCl_6$ (δ ($^{195}Pt$) = 0 ppm) in the case of $^{195}Pt$-NMR spectra. FAB(+) mass spectra were obtained on a ZAB-HSQ (V.G. Analytical Ltd.) spectrometer.

### 2.2. Synthesis of ligands

The ligands N,N-diphenyl-N′-benzoylthiourea (**HL$^1$**) and N,N-diphenyl-N′-(p-nitrobenzoyl) thiourea (**HL$^2$**) were prepared according to the reported methods /20,21/ as shown in Scheme 1. For the synthesis of **HL$^2$**, the used solvent was acetone instead of acetonitrile.

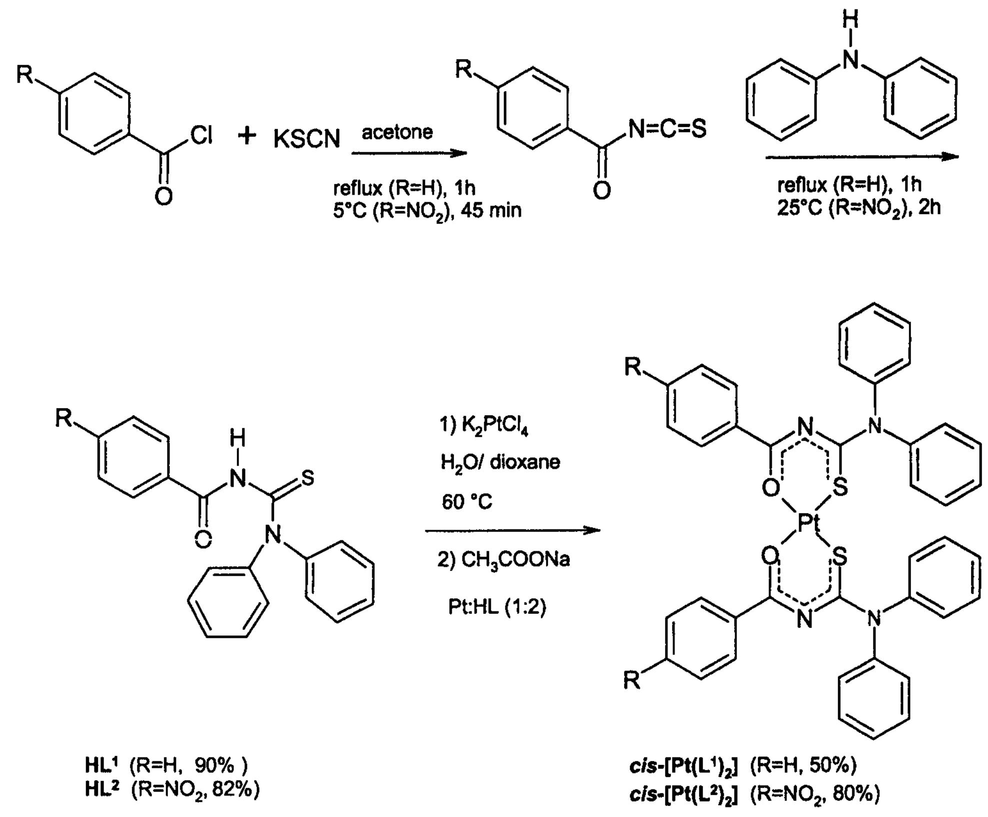

**Scheme 1:** Synthesis of the ligands N,N-diphenyl-N′-R-benzoylthiourea and their complexes *cis*-[$PtL_2$].

*2.2.1. N,N-diphenyl-N'-benzoylthiourea (HL[1])*

Pale yellow solid. Yield: 90 %, m.p. 122-124 °C. Anal. Calc. for $C_{20}H_{16}ON_2S$ (332.42 g/mol): C, 72.26%; H, 4.82%; N, 8.43%; S, 9.65%. Found: C, 71.89%; H, 4.97%; N, 8.51%; S, 9.58%. IR (KBr, $cm^{-1}$): ν(N-H) 3223 $cm^{-1}$ (m,br.), ν(C=O) 1692 $cm^{-1}$ (vs), ν(C=S) 1231 (vs). $^1$H-NMR ($CDCl_3$): δ 7.26 (m, 5H, $NPh_2$), 7.37 (m, 5H, $NPh_2$), 7.52 (t, $1H_{para}$,$2H_{meta}$, PhCO), 5.7.59 (d, $2H_{ortho}$, PhCO), 8.69 (s, $^1$H, NH).$^{13}$C-NMR ($CDCl_3$): δ 127.2 (s, 6C, $NPh_2$), 128.02 (s, 6C, $NPh_2$), 129.2 (s, 2C, PhCO), 129.7 (s, 2C, PhCO), 133.06 (s, 1C, PhCO), 133.3 (s, 1C, PhCO), 162.63 (s, 1C, C=O), 182.76 (s, 1C, C=S).

*2.2.2. N,N-diphenyl-N'-(p-nitrobenzoyl)thiourea (HL[2])*

Yellow crystals. Yield: 82 %, m.p. 168-170°C. Anal. Calc. for $C_{20}H_{15}O_3N_3S$ (377.42 g/mol): C, 63.65%; H, 4.00%; N, 11.13%; S, 8.49%. Found: C, 63.38%; H, 4.08%; N, 11.24%; S, 8.16%. IR (KBr, $cm^{-1}$): ν(N-H) 3150 $cm^{-1}$ (m), ν(C=O) 1699 $cm^{-1}$ (s), ν(C=S) 1247 (s). $^1$H-NMR ($CDCl_3$): δ 7.27 (m, 5H, $NPh_2$), 7.36 (m, 5H, $NPh_2$), 7.76 (d, $2H_{ortho}$ , $NO_2$-PhCO), 8.24 (d, $2H_{meta}$ , $NO_2$-PhCO), 8.67 (s, 1H, NH). $^{13}$C-NMR ($CDCl_3$):δ 127.2 (s, 6C, $NPh_2$), 128.2 (s, 4C, $NPh_2$), 129.2 (s, 2C, $NPh_2$), 124. 37 (s, 2C, $NO_2$-PhCO), 129.82 (s, 2C, $NO_2$-PhCO), 138.49 (s, 1C, $NO_2$-PhCO), 142.9 (s, 1C, $NO_2$-PhCO), 161.4 (s, 1C, C=O), 181.8 (s, 1C, C=S).

### 2.3. Synthesis of the platinum(II) complexes

*2.3.1. cis-bis(N,N-diphenyl-N'-benzoylthioureato) platinum(II) ( cis-[Pt(L[1])$_2$] )*

To a solution of HL[1] (0.17g, 0.50 mmol) in dioxane (40 mL) was added dropwise a solution of $K_2PtCl_4$ (0.10 g, 0.25 mmol) in water (20 mL), followed by sodium acetate (0.041 g, 0.5 mmol) in water (2 mL), and stirred for 2 h at 60°C . The reaction mixture was then stirred for 24 h at room temperature. The yellow precipitate was collected by filtration, washed several times with small portions of water, cold ethanol and dried in vacuo. Recrystallization of the yellow solid from hot dichloromethane gave small pale yellow crystals which were characterized by X-ray crystallography. Yield: 0.124 g (58.0%), m.p. 255-258 °C (dec.). Anal. Calc. for $C_{40}H_{30}O_2N_4S_2Pt$ (857.90 g/mol): C, 56.0%; H, 3.52%; N, 6.53%; S, 7.48%. Found: C, 55.66%; H, 3.80%; N, 6.58%; S, 7.27%. IR (KBr, $cm^{-1}$): ν(C=O) 1650 $cm^{-1}$ (m), ν(C=S) 1218 (m). FAB(+)-MS (matrix: 3-NBA): m/z 858.1 (M + , rel. int. 100%). $^1$H-NMR ($CDCl^3$) δ7.28 (m, 10H, $NPh_2$), 7.39 (m, 10H, $NPh_2$), 7.86 (d, $2H_{para}$,$4H_{meta}$, PhCO), 7.88 (d, $4H_{ortho}$, PhCO), 195 Pt-NMR ($CDCl_3$): -2583.6 ppm.

*2.3.2. cis-bis(N,N-diphenyl-N'-(p-nitrobenzoyl)thioureato) platinum(II) ( cis-[Pt(L[2])$_2$] )*

A similar procedure was carried out using HL[2] (0.19g, 0.50 mmol) and $K_2PtCl_4$ (0.10 g, 0.25 mmol) and stirring at room temperature for 2 days. Yield: 0.19 g (80.0%), m.p. 283-285 °C (dec.). Anal. Calc. for $C_{40}H_{28}O_6N_6S_2Pt$ (947.89 g/mol): C, 50.68%; H, 2.96%; N, 8.87%; S, 6.76%. Found: C, 50.3%; H, 3.05%; N, 8.54%; S, 6.38%. IR (KBr, $cm^{-1}$): ν(C=O) 1640 $cm^{-1}$ (m), ν(C=S) 1220 (w). FAB(+)-MS (matrix: 3-NBA): m/z 948.08 ($M^+$ , rel. int. 100%).$^1$H-NMR ($CDCl_3$): δ7.41 (m, 20H, $NPh_2$), 7.93 (d, $4H_{ortho}$ , $NO_2$-PhCO), 8.13 (d, $4H_{meta}$, $NO_2$-PhCO). $^{195}$Pt-NMR ($CDCl_3$): - 2426 ppm.

### 2.4. Crystal structure determinations

All single-crystal X-ray measurements were carried out on a CCD SMART APEX diffractometer equipped with a graphite monochromator using $MoK_\alpha$ radiation ($\lambda$ =0.71073 ). Data were collected at room temperature. The structures were solved by direct methods and refined against $F^2$ (all data, anisotropic thermal parameters for all non-H atoms, all H atoms located and fully refined with isotropic thermal parameters) using the SHELXS-97 and SHELXL-97 programs /22,23/. Crystal data collection and refinement details for ligand $HL^2$ and *cis*-[Pt($L^1$ )$_2$] complex are summarized in Table 1.

**Table 1**

Crystal data and refinement summary for $\mathbf{HL^2}$ and *cis*-**[Pt($L^1$)$_2$]**

| | $\mathbf{HL^2}$ | *cis*-**[Pt($L^1$)$_2$]** |
|---|---|---|
| Empirical formula | $C_{20}H_{15}O_3N_3S$ | $C_{40}H_{30}O_2N_4S_2Pt$ |
| Formula weight | 377.4 | 857.9 |
| Temperature (K) | 297(2) | 297(2) |
| Crystal size (mm) | 0.3 x 0.2x 0.25 | 0.25 x 0.2 x 0.2 |
| Crystal system | Triclinic | Triclinic |
| Space group | P-1 | P1 |
| Unit cell dimensions | | |
| a (Å) | 6.865(1) | 10.0630(6) |
| b (Å) | 10.098(1) | 11.2513(6) |
| c (Å) | 13.401(2) | 16.4199(9) |
| $\alpha$ (°) | 88.858(2) | 105.562(1) |
| $\beta$ (°) | 77.841(2) | 91.442(1) |
| $\gamma$ (°) | 88.798(3) | 104.217(1) |
| Volume (Å$^3$) | 907.8(2) | 1727.8(2) |
| Z | 2 | 2 |
| Densidad(calc.) (g/cm$^3$) | 1.381 | 1.649 |
| $\mu$(Mo-$K_\alpha$)/ (mm$^{-1}$) | 0.204 | 4.223 |
| F(000) | 392 | 848 |
| 2 $\theta$ Range (°) | 3.10 - 55.94 | 3.90-56.0 |
| Collected reflections | 3779 | 8551 |
| Observed reflections | 2883 | 7787 |
| Refined parameters | 248 | 883 |
| $R_{int}$ | 0.0149 | 0.0140 |
| Final R1/wR2 [I>2 $\sigma$ (I)] | 0.0571 / 0.1261 | 0.0289 / 0.0675 |
| (all data) | 0.0759 / 0.1370 | 0.0331 / 0.0698 |
| Goodness of fit on $F^2$ | 1.08701 | 1.0310 |
| Greatest difference peak and hole (e Å$^{-3}$) | 0.256 / -0.183 | 1.375 / -0.516 |

### 2.5. Biological evaluation

### *2.5.1. Tumor cells culture*

The mouse mammary adenocarcinoma TA3 used in this study was obtained from ascites fluid of young adult male $CAF^1$ Jax mice, which was cultured at 37 °C in Dulbecco's modified Eagle medium (DMEM) (Sigma Chemical Co.) supplemented with 10% fetal calf serum (FBS) (Difco, Detroit, MI), 25 mM HEPES, 44 mM sodium bicarbonate, 100 U $mL^{-1}$ penicillin and 100 μg $mL^{-1}$ streptomycin.

### *2.5.2. Inhibition of the TA 3 cell line growth*

For these assays, 1.8 - 2.2 x 10 5 cells $mL^{-1}$ were seeded in 20 mL of culture medium during 96 h. After 24 h of the seeding, either the ligand $HL^1$ or $HL^2$ (20, 40 and 60 μM) or their complexes *cis*-$[PtL_2]$ (1, 5 and 10 μM) in DMSO was added to every culture. Parallel cultures were used as control. Cell numbers were determined using a Neubauer counting chamber every 24 h. The concentrations of every compound were plotted with respect to the percentage of cell survival. The $IC_{50}$ values were obtained by graphical interpolation at 48 h of time of exposure on each one of the compounds. These values represent the drug concentration (μM) required to inhibit cell growth by 50%. All assays were performed in triplicate and repeated three times in independent pattern /24/.

### 3. RESULTS AND DISCUSSION

### 3.1. Synthesis and spectroscopic characterization of the ligands and their platinum(II) complexes

The ligands N,N-diphenyl-N'-benzoylthiourea (**$HL^1$**) and N,N-diphenyl-N'-(p-nitrobenzoyl) thiourea (**$HL^2$**) were prepared according the methods described by Hartmann *et al.* and Brindley *et al.*, respectively /20,21/. The synthesis involves the reaction of 4-R-benzoyl chloride with potassium thiocyanate in acetone followed by reacting 4-R-benzoyl isothiocyanate with diphenylamine (Scheme 1). The ligands $HL^1$ and $HL^2$ were obtained in satisfactory yields (82-90 %) and characterized by elemental analysis and IR, $^1$H-NMR and $^{13}$C-NMR spectroscopy. The molecular structure of $HL^2$ has been determined by X-ray diffraction.

The complexes *cis*-**$[Pt(L^1)_2]$** and *cis*-**$[Pt(L^2)_2]$** were prepared by reacting $K_2PtCl_4$ with $HL^1$ or $HL^2$ (Pt:HL 1:2) in dioxane/water solution at 60 °C. The complex *cis*-$[Pt(L^1)_2]$ was recrystallized from dichloromethane to yield suitable single crystals and its structure has been confirmed by X-ray diffraction. The complexes *cis*-$[PtL_2]$ were characterized by elemental analysis and IR, FAB(+)-mass, $^1$H-NMR and $^{195}$Pt-NMR spectroscopy.

In the IR spectra , the N-H stretching vibrations assigned at 3150-3220 $cm^{-1}$ for ligands **$HL^1$** and **$HL^2$** disappeared upon complexation due to the deprotonation of (NHCO) amide group in the ligands. The strong absorption bands 1692-1696 $cm^{-1}$ and 1231-1247 $cm^{-1}$ corresponding to the vibrations ν(C=O ) and ν(C=S), respectively, are shifted to lower frequencies 1640-1650 $cm^{-1}$ and 1218-1220 $cm^{-1}$ respectively, upon coordination, which proves that the deprotonated ligands are coordinated to platinum(II) ion through oxygen and sulfur donor atoms /25-27/.

In the $^1$H-NMR spectra, the deprotonation of the ligands in the complexes is confirmed by the disappearance of the N-H signal present in the ligands **HL**[1] and **HL**[2] in the range of 8.67- 8.69 ppm. For the ligand **HL**[2], the aromatic protons signals were affected by the presence of nitro substituent group in the *para* position of benzoyl moiety. These signals are shifted to downfield for the protons in the *meta* ( 0.72 ppm) and *ortho* (0.17 ppm) positions relative to unsubstituted benzoyl moiety of ligand **HL**[1].

The $^{195}$Pt-NMR spectra of the complexes *cis*-**[Pt(L$^1$)$_2$]** and *cis*-**[Pt(L$^2$)$_2$]** showed a single $^{195}$Pt resonance at -2583.6 and -2426.0 ppm, respectively. These results confirm the presence of only one isomer which exists in $CDCl_3$ solution for both complexes, and is in agreement with the $^{195}$Pt chemical shift range found for other *cis*-**[PtL$_2$]** square planar platinum(II) complexes with ligands N,N-dialkyl-N′-acylthiourea described in the literature /26, 28/. The nitro group effect in the complex *cis*-**[Pt(L$^2$)$_2$]** is observed for the $^{195}$Pt chemical shift of about 158 ppm to downfield relative to complex *cis*-**[Pt(L$^1$)$_2$]**, and this evidence demonstrates the sensitivity of the $^{195}$Pt nucleus to electronic changes on the benzoyl moiety which originate a decrease of the electron density at the platinum centre /26, 29/.

To confirm the proposed structures of the ligands and their platinum(II) complexes, structural determinations of single crystal were carried out for the ligand HL[2] and complex *cis*-**[Pt(L$^1$)$_2$]**.

### 3.2. Structural data

The molecular structures of **HL**[2] and *cis*-**[Pt(L$^1$)$_2$]** are shown in Figures 1 and 2, respectively, whereas their selected bond lentghs and bond angles are presented in Table 2. The crystal structure of the ligand N,N-diphenyl-N′-(p-nitrobenzoyl)thiourea **HL**[2] (Fig. 1) shows a twisted conformation with respect to the carbonyl and thiocarbonyl moieties where the O(1) and S(1) atoms are pointing in opposite directions as reflected by the torsion angles O(1)-C(1)-N(1)-C(2) and C(1)-N(1)-C(2)-S(1) of 5.74 and -132.58 °, respectively. The C(1)-O( 1) bond (1.209(3) Å) is typical for a C=O double bond (1.23 Å) while the C(2)-S(1) length (1.664 (2) Å) suggests a bond order between a single and double bond in the (C=S) thiocarbonyl moiety. The angle formed by the phenyl rings bounded to N(2) is 115.4(4)° (almost perpendicular) due to the steric interaction between their aromatic protons. The molecules of **HL**[2] pack in stacks of alternating orientation and adjacent molecules are bonded by intermolecular hydrogen bond between the N-H amide and thiocarbonyl moiety N(1)-H ···· S(1) ( N(1) ··· S(1) 3.406 Å, H(1) ··· S(1) 2.612 Å, N(1)-H(1) ... S(1) 154.03 ° ).

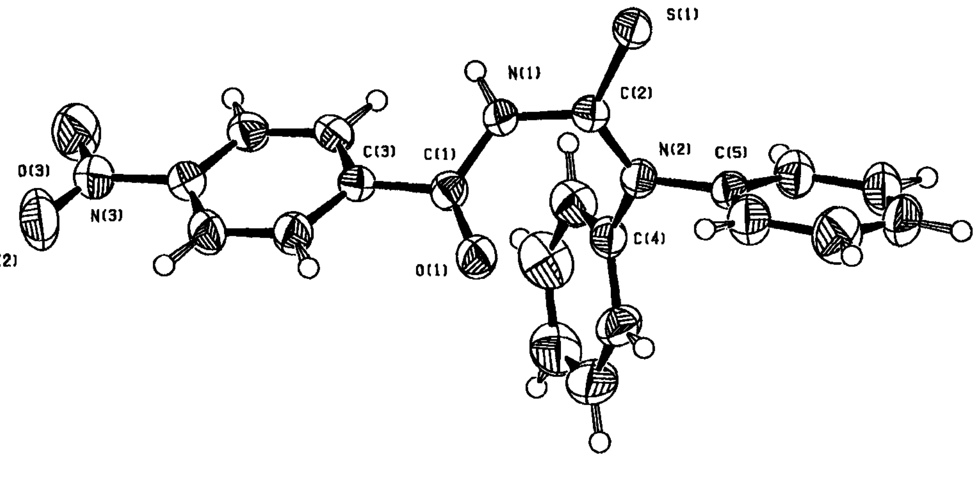

**Fig. 1:** Molecular structure of HL$^2$ (50 % thermal ellipsoids).

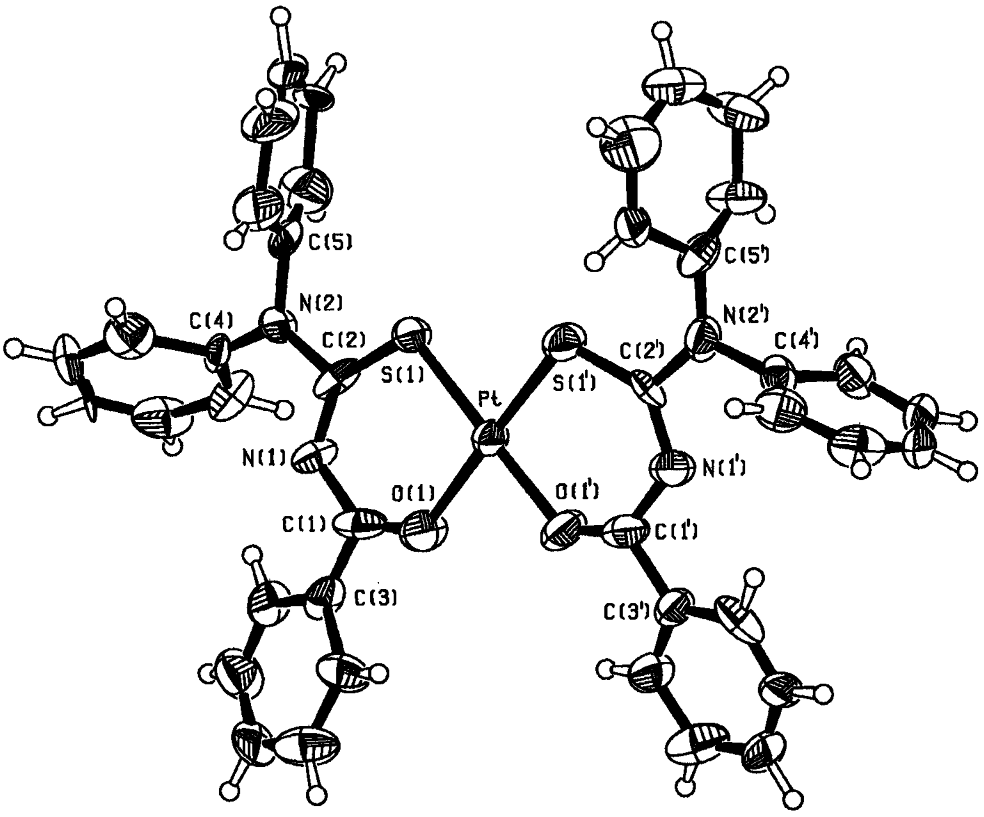

**Fig. 2:** Molecular structure of *cis*-[Pt($L^1$)$_2$] (50 % thermal ellipsoids).

The molecular structure of complex ***cis*-[Pt($L^1$)$_2$]** (Fig. 2) shows a nearly square-planar geometry (torsion angles Pt-O(1)-C(1)-N(1) 4 °, Pt-S(1)-C(2)-N(1) -6.6 °) with two deprotoned ligands $L^1$ coordinated to platinum(II) ion through the oxygen and sulfur donor atoms in a *cis* arrangement, similar to the *cis*-[$PtL_2$] complexes with ligands N,N-diethyl-N'-benzoylthiourea and N,N-di(n-butyl)-N'-benzoylthiourea /14, 26/. By comparison with the structure of the free ligand **$HL^2$**, the bond lengths of the thiocarbonyl moieties [C(1)-S(1) 1.74(2), C(1')-S(1') 1.76(1) Å] and carbonyl [C(1)-O(1)$^1$.31(2), C(1')-O(1') 1.37(2) Å] in the complex *cis*-[Pt($L^1$)$_2$] are on average longer (1.75 and 1.34 Å, respectively) than those in the ligand **$HL^2$** (C=S 1.664(2) and C=O 1.209(3) Å, respectively) while the corresponding two contiguous C-N bond lengths (N(1)-C(1) and N(1)-C(2)) of the complex *cis*-[Pt($L^1$)$_2$] are on average shorter (1.25 and 1.36 Å, respectively) compared to **$HL^2$** (N(1)-C(1) 1.389(3), N(1)-C(2) 1.391(3) Å). These results confirm the decrease of the bond orders of the carbonyl and thiocarbonyl groups upon complexation and, together with the changes in the C-N bond lengths, indicate the presence of an extensive delocalization of electron density in the chelate ring of the complex ***cis*-[Pt($L^1$)$_2$]**; furthermore this is confirmed by IR, $^1$H-NMR and $^{195}$Pt-NMR spectroscopy.

**Table 2**

Selected bond lengths (Å) and bond angles (°) for $HL^2$ and *cis*-$[Pt(L^1)_2]$

| | $HL^2$ | *cis*-$[Pt(L^1)_2]$ | |
|---|---|---|---|
| | | ligand $L^1$ | ligand $L^{2'}$ |
| *Bond lengths* | | | |
| S(1)-C(2) | 1.664(2) | 1.74(2) | 1.76(1) |
| O(1)-C(1) | 1.209(3) | 1.31(2) | 1.37(2) |
| N(1)-C(1) | 1.389(3) | 1.25(2) | 1.24(2) |
| N(1)-C(2) | 1.391(3) | 1.36(2) | 1.35(2) |
| N(2)-C(2) | 1.34(3) | 1.33(2) | 1.28(2) |
| N(2)-C(4) | 1.447(3) | 1.39(2) | 1.52(2) |
| N(2)-C(5) | 1.453(3) | 1.50(2) | 1.44(2) |
| C(1)-C(3) | 1.492(3) | 1.50(2) | 1.53(2) |
| Pt-O(1) | 2.06(1) | 1.961(1) | |
| Pt-S(1) | 2.235(5) | 2.235(5) | |
| *Bond angles* | | | |
| O(1)-C(1)-N(1) | 123.0(2) | 130.0(2) | 127.0(1) |
| C(1)-N(1)-C(2) | 124.4(2) | 134.0(2) | 135.0(1) |
| N(1)-C(2)-S(1) | 120.1(2) | 126.0(1) | 122.0(1) |
| N(1)-C(2)-N(2) | 116.0(2) | 115.0(1) | 122.0(1) |
| C(2)-N(2)-C(5) | 120.5(2) | 118.0(1) | 127.0(1) |
| C(2)-N(2)-C(4) | 123.9(2) | 119.0(1) | 122.0(1) |
| C(4)-N(2)-C(5) | 115.4(4) | 123.0(1) | 112.0(1) |
| O(1)-C(1)-C(3) | 122.2(2) | 108.0(1) | 114.0(1) |
| N(1)-C(1)-C(3) | 114.9(2) | 122.0(2) | 118.0(1) |
| N(2)-C(2)-S(1) | 123.8(2) | 119.0(1) | 116.0(1) |
| C(2)-S(1)-Pt(1) | | 107.9(6) | 110.5(5) |
| C(1)-O(1)- Pt(1) | | 125.0(1) | 129.0(1) |
| O(1)-Pt-S(1) | | 96.6(3) | 94.8(4) |
| O(1)-Pt-O(1′) | | 81.7(4) | |
| S(1)-Pt-S(1′) | | 86.9(2) | |
| O(1)-Pt-S(1′) | | 176.3(4) | |
| O(1′)-Pt-S(1) | | 176.6(4) | |

### 3.3. Antitumor evaluation

The cytotoxic activity of the acylthiourea ligands and their platinum (II) complexes were evaluated on TA3 tumor cell line. Figures 3, 4 and 5 show the concentration-dependent inhibitory effects for $HL^1$, $HL^2$, *cis*-$[Pt(L^1)_2]$ and *cis*-$[Pt(L^2)_2]$ on the growth of TA3 tumor cell line, respectively. In Figure 3, it can be

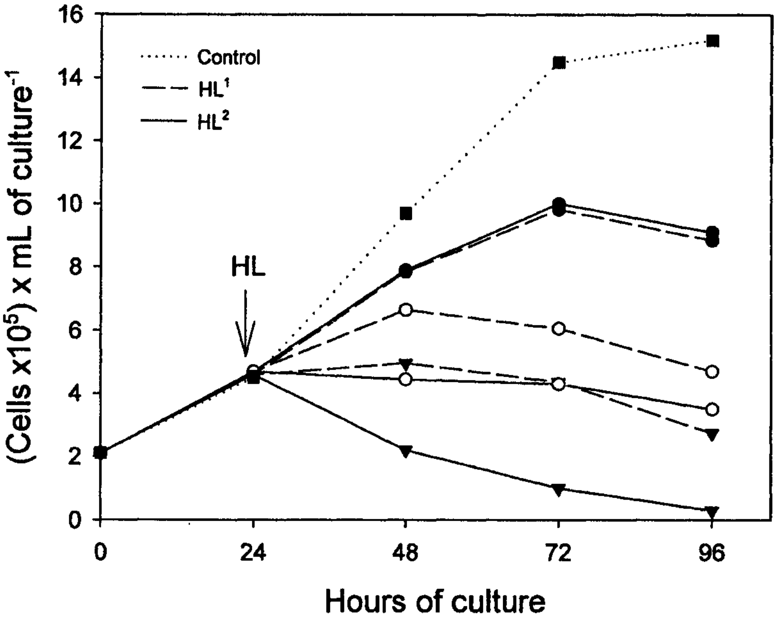

**Fig. 3:** The effect of $HL^1$ and $HL^2$ ligand concentrations on the culture growth of TA3 tumor cell line: (●) 20 μM, (o) 40 μM and (▼) 60 μM. Each value is the mean ± SD of three independent experiments with each assay performed in triplicate.

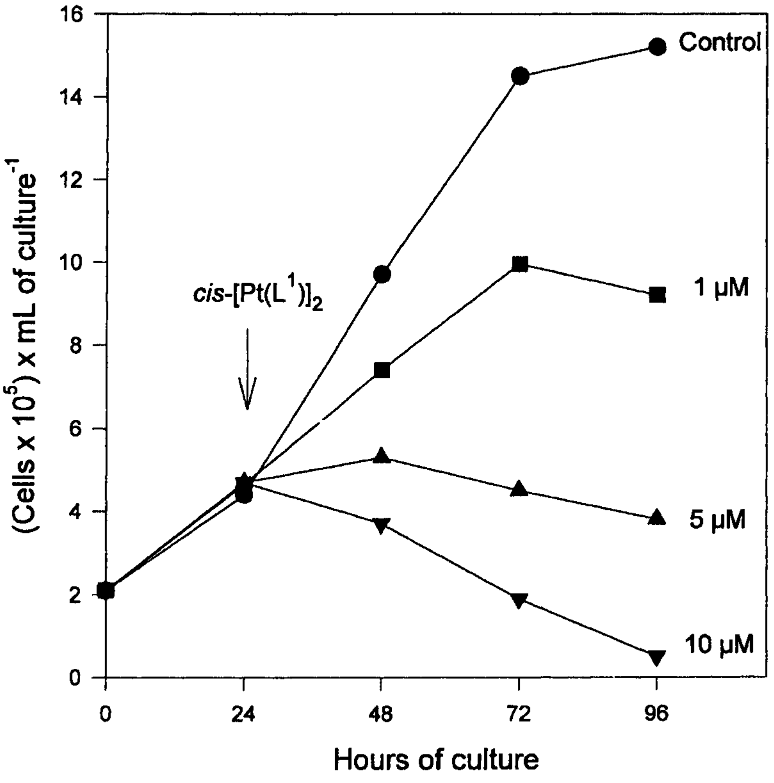

**Fig. 4:** The effect of the concentration of *cis*-$[Pt(L^1)_2]$ complex on TA3 tumor cell line growth in culture. Each value is the mean of three independent experiments with each assay performed in triplicate.

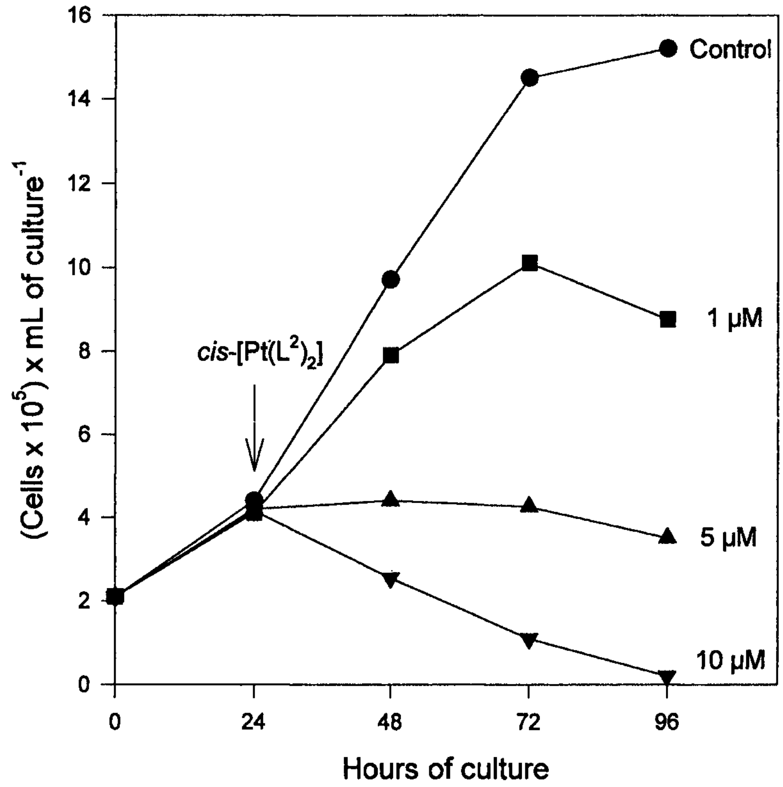

**Fig. 5:** The effect of *cis*-$[Pt(L^2)_2]$ complex concentration on TA3 tumor cell line growth in culture. Each value is the mean of three independent experiments with each assay performed in triplicate.

observed that the ligand $HL^2$ is more cytotoxic than $HL^1$ in the range of 40-60 μM while *cis*-$[Pt(L^1)_2]$ and *cis*-$[Pt(L^2)_2]$ exhibited higher cytotoxic activities than the ligands (Figs. 4 and 5). A similar level of cellular growth inhibition was obtained for *cis*-$[Pt(L^2)_2]$ and $HL^2$ in the range of 1-10 and 20-60 μM, respectively. A strong inhibition of the cell proliferation was shown by the complexes *cis*-$[Pt(L^1)_2$ and *cis*-$[Pt(L^2)_2]$ at the concentration of 10 μM, where the toxicity was drastically increased and the cell survival was completely inhibited at 72 h of exposure. These results indicate that the cytotoxic activity is enhanced when the ligands $HL^1$ or $HL^2$ are coordinated to Pt(II). Probably the *cis*-$[PtL_2]$ complexes can be easily transported through cellular membrane as a result of their high hydrophobicity and thus exert their cytotoxicity on cellular DNA.

Figure 6 shows the cytotoxic effect for the *cis*-$[Pt(L^1)_2]$ and *cis*-$[Pt(L^2)_2]$ complexes with respect to the ligands $HL^1$ and $HL^2$ on the percentage of cell survival at 48 h of exposure on culture medium. While the ligand $HL^2$ was only about 20 % more cytotoxic than $HL^1$, *cis*-$[Pt(L^1)_2]$ and *cis*-$[Pt(L^2)_2]$ were much more cytotoxic than their respective ligands $HL^1$ or $HL^2$. This demonstrates that the presence of a nitro group on the benzoyl moiety may be important in some degree on the growth inhibition of the TA3 tumor cell line for $HL^2$, whereas in the cytotoxicity of the platinum (II) complexes, the participation of nitro group is slightly relevant, and rather the hydrophobicity of these *cis*-$[PtL_2]$ complexes plays an important role in the cytotoxic activity /30-32/.

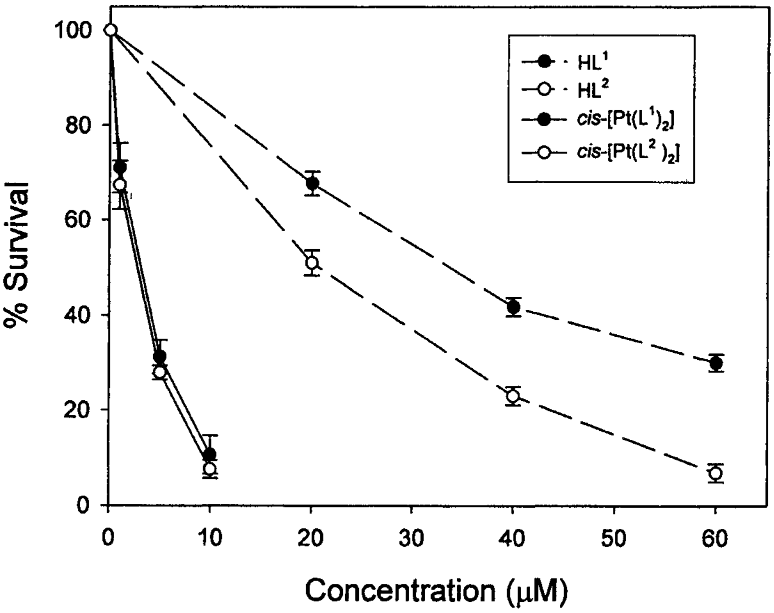

**Fig. 6:** Cytotoxic effect of the ligands $HL^1$, $HL^2$ and their complexes *cis*-$[Pt(L^1)_2]$ and *cis*-$[Pt(L^2)_2]$ on TA3 tumor cell line cultured during 48 h. Each value is the mean ± SD of three independent experiments, where each assay was performed in triplicate.

A comparison of $IC_{50}$ values for the tested compounds is provided in Table 3. The ligand $\mathbf{HL^2}$ with a nitro substituent group showed greater cytotoxicity (34.9 μM) compared to $\mathbf{HL^1}$ (23.1 μM), whereas the cytotoxic activities of their *cis*-$[PtL_2]$ complexes were about ten times higher than their respective ligands. The $IC_{50}$ values of the ***cis*-$[Pt(L^1)_2]$** and ***cis*-$[Pt(L^2)_2]$** complexes (2.8 and 2.6 μM, respectively) turned out to be slightly better than cisplatin ($IC_{50}$ = 4.2 ± 1.2 μM), evaluated on oesophageal tumor cells (carcinoma) /33/. These results demonstrate that the bis-chelate ***cis*-$[Pt(L^1)_2]$** and ***cis*-$[Pt(L^2)_2]$** complexes, with a square-planar geometry play an important role in the inhibition of TA3 tumor cell growth.

**Table 3**

Cytotoxic activity of $\mathbf{HL^1}$, $\mathbf{HL^2}$ and their ***cis*-$[PtL_2]$** complexes against to TA3 tumor cell line.

| Compound | $IC_{50}$ (μM)[a] |
|---|---|
| $HL^1$ | 34.9 ±1.4 |
| *cis*-$[Pt(L^1)_2]$ | 2.8 ± 0.3 |
| $HL^2$ | 23.1 ±1.3 |
| *cis*-$[Pt(L^2)_2]$ | 2.6 ± 0.1 |

[a] $IC_{50}$ corresponds to the concentration required to inhibit 50 % of the culture growth when cells are exposed to compounds for 48 h. Each value is the mean ± SD of three independent experiments with each assay performed in triplicate.

In summary, we have prepared the bis-chelate complexes ***cis*-[Pt($L^1$)$_2$]** and ***cis*-[Pt($L^2$)$_2$]**, which are more cytotoxic on the TA3 tumor cell line at micromolar concentration compared to acylthiourea ligands, **$HL^1$** and **$HL^2$** . Moreover, it was demonstrated that the participation of the nitro substituent group in the *para* position of benzoyl moiety in **$HL^2$** slightly increases its cytotoxic activity with respect to ligand **$HL^1$**, whereas, in comparison to complex activities, this substitution is irrelevant. These results could represent an important contribution to establish some mechanism of action of bis-chelate complexes, *cis*-[$PtL_2$] (not analogous to cisplatin) related to cellular DNA.

### 4. SUPPLEMENTARY MATERIAL

Crystallographic data for the structure analysis have been deposited with the Cambridge Crystallographic Data Center as supplementary publication numbers CCDC 214453 and 214454. Copies of the data can be obtained free of charge from the Director, CCDC, 12 Union Road, Cambridge, CB2 1EZ, UK (fax: +44-1223-336033; e-mail: deposit@ccdc.cam.ac.uk).

### ACKNOWLEDGEMENTS

W. H. and J.C.M would like to thank DAAD for the doctoral scholarship granted in Chile. This study also was supported by Departamento de Post-grado de la Universidad de Chile (PG/91)

### REFERENCES

1. B. Rosenberg, L. Van Camp, J. Krosko and V. Mansour, *Nature*, **222**, 385 (1969).
2. M. Nicolini and L. Sindellari. *Lectures in bioinorganic chemistry*. Ed. Cortina Raven, Verona, Italy, pp.25-30 (1991).
3. B. Keppler, *Metal Complexes in Cancer Chemotherapy*, Ed. VCH, Heidelberg, Germany, pp. 27-32 (1993).
4. U. Bierbach, M. Sabat and N. Farrell, *Inorg. Chem*., **39**, 1882 (2000).
5. J. Reedijk, *Chem. Commun*., 801 (1996).
6. S. Bruhn, J. Toney and S. Lippard, *Prog. Inorg. Chem*., *Bioinorg. Chem*., **38**, 477 (1991).
7. E. Jamieson and S. Lippard, *Chem. Rev*., **99**, 2467 (1999).
8. J. Hickman, *Cancer Metastasis Rev*., **11**, 121 (1992).
9. N. Madias and J. Harrington, *Am. J. Med*. , **65**, 307 (1978).
10. M. Kartalou and J. Essigmann, *Mutat. Res*. , **478**, 1 (2001).
11. U. Bierbach, T. Hambley, J. Roberts and N. Farrell, *Inorg. Chem*., **35**, 4865 (1996).
12. U. Bierbach, J. Roberts and N. Farrell, *Inorg. Chem*., **37**, 717 (1998).
13. E. Martins, H. Baruah, J. Kramarczyk, G. Saluta, C. Day, G. Kucera and U. Bierbach, *J. Med. Chem*., **44**, 4492 (2001).

14. C. Sacht, M. Datt, S. Otto and A. Roodt, *J. Chem. Soc. Dalton Trans.*, 727 (2000).
15. C. Sacht, M.S. Datt, S. Otto and A. Roodt, *J. Chem. Soc. Dalton Trans.*, 4579 (2000).
16. C. Sacht and M.S. Datt, *Polyhedron,* **19**, 1347 (2000).
17. L. Beyer, E. Hoyer, H. Henning and R. Kirmse, *J. Prakt. Chem.*, **377**, 829 (1975).
18. R. Richter, L. Beyer and J. Kaiser, *Z. Anorg. Allg. Chem.*, **461**, 67 (1980).
19. A. Rodger, K. Patel, K. Sanders, M. Datt, C. Sacht and M. Hannon, *J. Chem. Soc. Dalton Trans.*, 3656 (2002).
20. H. Hartmann and I. Reuther, *J. Prakt. Chem.*, **315**, 144 (1973).
21. J. Brindley, J. Caldwell, G. Meakins, S. Plackett and S. Price, *J. Chem. Soc. Perkin* **I**, 1153 (1987).
22. G. M. Sheldrick, SHELXS 97, Program for Solving Crystal Structures, University of Göttingen, 1997.
23. G. M. Sheldrick, SHELXL 97, Program for Structure Refinement, University of Göttingen, 1997.
24. J. Ferreira, L. Coloma, E. Fones, M. Letelier, Y. Repetto, A. Morello and J. Aldunate, *FEBS Lett.,* **234**, 485 (1988).
25. L. Beyer, E. Hoyer, L. Liebscher and H. Hartmann, *Z. Chem.*, **21**, 81 (1980).
26. K.R. Koch, A. Irving and M. Matoetoe, *Inorg. Chim. Acta*, **206**, 193 (1993).
27. K.R. Koch, C. Sacht and S. Bourne, *Inorg. Chim. Acta*, **232**, 109 (1995).
28. K.R. Koch, T. Grimmbacher and C. Sacht, *Polyhedron*, **17**, 267 (1995).
29. P. Pregosin, *Coord. Chem. Rev.,* **44**, 247 (1982).
30. M. Hall and T. Hambley, *Coord. Chem. Rev.,* **232**, 49 (2002).
31. T. Okada, I. El-Mehasseb, M. Kodaka, T. Tomohiro, K. Okamoto and H. Okuno, *J. Med. Chem.*, **44**, 4661 (2001).
32. Y. Sang, R. Song, D. Hyun, M. Jin and Y. Soo, *Bioorg. Med. Chem.*, **11**, 1753 (2003).
33. D. Sheehan and G. Meade, *Biochem. Soc. Trans.*, **28**, 27 (2000).

